# An Efﬁcient Synthesis of 1-Aryl-3-(indole-3-yl)-3-(2-aryl-1,2,3-triazol-4-yl)propan-1-one Catalyzed by a Brønsted Acid Ionic Liquid

**DOI:** 10.3390/molecules15129197

**Published:** 2010-12-10

**Authors:** Chen-Jiang Liu, Chuan-Ji Yu

**Affiliations:** 1 Physics and Chemistry Detecting Center, Xinjiang University, 830046 Urumqi, China; 2 Key Laboratory of Oil and Gas Fine Chemicals Ministry of Education, School of Chemistry and Chemical Engineering, Xinjiang University, 830046 Urumqi, China; E-Mail: cjxyycj@126.com (C-J.Y.)

**Keywords:** Brønsted acidic ionic liquid, Michael reaction, indoles, 1,2,3-triazole

## Abstract

An efficient synthesis of novel 1-aryl-3-(indole-3-yl)-3-(2-aryl-1,2,3-triazol-4-yl)propan-1-ones from indoles and 1-aryl-3-(2-aryl-1,2,3-triazol-4-yl)propan-1-one using a Brønsted acid ionic liquid [Sbmim][HSO_4_] as catalyst is described. Satisfactory results with excellent yields and short reaction time were obtained in the experiments. The catalyst could be recovered conveniently and reused efficiently.

## Introduction

In recent years, *β*-indolylketones have received much attention because they represent an important substructure in both biologically active compounds and natural products [1]. A simple and direct approach for their synthesis involves the conjugate addition of indole and *α,β*-unsaturated ketones in the presence of either protic or Lewis acids. During the last decade, several improved methods have been reported for the preparation of these compounds using various catalysts such as FAP [2], I_2_ [3], Zn-HAP [4], CAN [5], InBr_3_ [6], PTSA [7], Bi(NO_3_)_3_ [8], HfCl_4_ and ScCl_3_ [9], PVSA [10], pyrrolidine and HClO_4_ [11], GaI_3_ [12], Zr(OTf)_4 _ [13], Bi(OTf)_3_ [14] and so on. However, several of these reported procedures suffer from the drawbacks such as strong acidic conditions, long reaction times, complex handling and low yields of products. Hence, new efficient and green procedures are still in strong demand. Recently, ionic liquids (ILs) have been widely used as environmentally benign reaction media in organic synthesis owing to their unique properties of nonvolatility, nonflammability, and recyclability [15,16]. In particular, the synthesis of task-speciﬁc ILs with special functions according to the requirements of a speciﬁc reaction has become an attractive ﬁeld [17,18]. Recently, Yang *et al*. [19] reported the use of the Brønsted acid ionic liquid [Sbmim][HSO_4_] as catalyst for the hydrolysis of soybean isoflavone glycosides. In this process [Sbmim][HSO_4_] has good catalytic activities which are similar to those of sulfuric acid, giving a conversion of glycitin of more than 90%. 

**Scheme 1:**
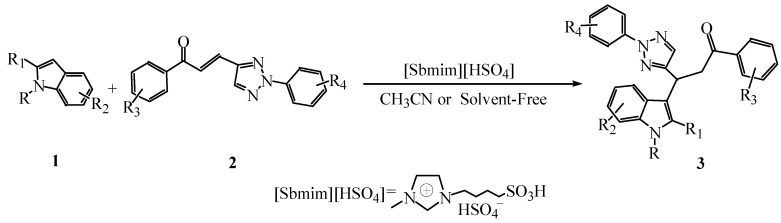
The synthesis of 1-aryl-3-(indol-3-yl)-3-(2-aryl-1,2,3-triazol-4-yl)propan-1-ones catalyzed by the ionic liquid [Sbmim][HSO_4_].

Due to their unique biological properties, 1,2,3-triazole derivatives have attracted much attention [20]. In this work, we studied the possibility to synthesize 1-aryl-3-(indol-3-yl)-3-(2-aryl-1,2,3-triazol-4-yl)propan-1-ones using 1-aryl-3-(2-aryl-1,2,3-triazol-4-yl)propan-1-one as substrates instead of ordinary *α,β*-unsaturated ketones and employing the Brønsted acid ionic liquid [Sbmim][HSO_4_] as catalyst (Scheme 1). Herein, an efficient and practical method for the synthesis of target compounds is described and none of them has been reported yet in the literature.

## Results and Discussion

Initially, to evaluate the effect of the catalyst [Sbmim][HSO_4_] under different reaction conditions, the reaction of indole and 1-phenyl-3-(2-phenyl-1,2,3-triazol-4-yl)propan-1-one was selected as a model reaction. The results were presented in Table 1. It was clear that the best solvent was acetonitrile and the best molar ratio of IL/substrate is 10% (Table 1, entry 3). The influence of the reaction time on the yield was also investigated as shown in Table 1, entries 3, 8–12. It was found that a higher yield occurred when the reaction time was 3 h (Table 1, entry 11), although, the yield did not change signiﬁcantly when the reaction time was increased from 1 h to 5 h. For the purpose of saving energy, we chose 1 h as the reaction time. Hence, the best conditions employed a 0.1:1:1 mole ratio of [Sbmim][HSO_4_], indole and 1-phenyl-3-(2-phenyl-1,2,3-triazol-4-yl)propan-1-one at 80 °C for 1 h using acetonitrile as solvent.

The recycling performance of TSIL [Sbmim][HSO_4_] was also investigated in the reaction of indole and 1-phenyl-3-(2-phenyl-1,2,3-triazol-4-yl)propan-1-one. After the separation of product, the filtrate containing catalyst was distilled under vacuum to remove water and the resulting catalyst was reused directly for the next run. 

**Table 1:** Effect of the catalyst[Sbmim][HSO_4_] in the reaction of indole and 1-phenyl-3-(2-phenyl-1,2,3-triazol-4-yl)propan-1-one under different conditions. ^a^

Entry	Solvent	[Sbmim][HSO_4_] (mol %)	Time (h)	Yield (%)^b^
1	Ethyl acetate	10	4	70
2	Methanol	10	4	87
3	Acetonitrile	10	4	95
4	Acetonitrile	none	4	0
5	Acetonitrile	2.5	4	79
6	Acetonitrile	5	4	91
7	Acetonitrile	15	4	94
8	Acetonitrile	10	0.5	79
9^c^	Acetonitrile	10	1	95, 92, 90
10	Acetonitrile	10	2	98
11	Acetonitrile	10	3	99
12	Acetonitrile	10	5	97

^a ^Reaction conditions: indole (2 mmol), 1-phenyl-3-(2-phenyl-1,2,3-triazol-4-yl)propan-1-one (2 mmol) and catalyst in solvent (10 mL), 80 °C; ^b ^Isolated yield; ^c ^catalyst was recycled three times.

As shown in Table 1, Brønsted acidic ionic liquid [Sbmim][HSO_4_] can be recycled at least three times without any significant decrease in catalytic activity, the yields ranged from 95% to 90% (entry 9^c^). This indicated that the ionic liquid [Sbmim][HSO_4_] was an eﬃcient and recyclable catalyst for the reaction. In order to check the generality of the procedure, a variety of substituted indoles were reacted with 1-aryl-3-(2-aryl-1,2,3-triazol-4-yl)propan-1-one. In general, the reaction proceeded easily under the optimum conditions described above and the adducts were isolated in excellent yields (Table 2). 

**Table 2:** [Sbmim][HSO_4_]-catalyzed synthesis of 1-aryl-3-(indole-3-yl)-3-(2-aryl-1,2,3-triazol-4-yl)propan-1-one.

Entry	R	R_1_	R_2_	R_3_	R_4_	Mp (°C)^a^	Yields (%)^b^	
							A^c^	B^c^
**3a**	H	H	H	H	H	148–150	93	89
**3b**	H	H	H	4-CH_3_	H	157–159	98	97
**3c**	H	H	H	4-OCH_3_	H	188–191	97	92
**3d**	H	H	H	4-Cl	H	140–143	95	96
**3e**	H	H	H	4-Br	H	143–146	96	93
**3f**	H	H	5-Br	2,4-Cl_2_	H	144–147	93	87
**3g**	H	CH_3_	H	H	H	163–165	97	95
**3h**	H	CH_3_	H	H	4-Br	165–168	89	86
**3i**	H	CH_3_	H	2-Cl	H	142–144	97	94
**3j**	CH_3_	H	H	4-OCH_3_	H	136–138	95	97

^a^ Melting points were uncorrected;^ b^ Isolated yield;^ c^ Method A: [Sbmim][HSO_4_] (0.02 mmol), 1-aryl-3-(2-aryl-1,2,3-triazol-4-yl)propan-1-one (2 mmol), indoles (2 mmol), acetonitrile (10 mL), 80 °C, 1 h; Method B: [Sbmim][HSO_4_] (0.02 mmol), 1-aryl-3-(2-aryl-1,2,3-triazol-4-yl)propan-1-one (2 mmol), indoles (2 mmol), solvent-free, 90 °C, 1 h.

The results obtained indicated that the electron donating or withdrawing groups at the indole ring did not seem to affect the reaction signiﬁcantly in terms of yields. As environmental consciousness in chemical research and industry has increased, the challenge for a sustainable environment has called for clean procedures that can avoid the use of organic solvents. Hence, we also examined the reaction of indoles and 1-aryl-3-(2-aryl-1,2,3-triazol-4-yl)propan-1-one under solvent-free condition, and the products were obtained in excellent yields (Table 2).

**Scheme 2:**
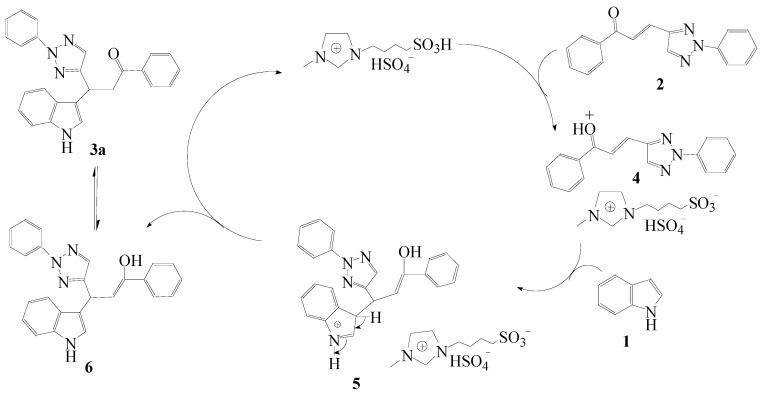
The proposed mechanism of synthesizing *β*-indolyketones catalyzed by ionic liquid [Sbmim][HSO_4_].

A proposed reaction mechanism for the conjugate addition of indole to 1-phenyl-3-(2-phenyl-1,2,3-triazol-4-yl)propan-1-one is presented in Scheme 2. The catalyst [Sbmim][HSO_4_] coordinates with the oxygen atom of 1-phenyl-3-(2-phenyl-1,2,3-triazol-4-yl)propan-1-one (**2**) to give intermediate **4**. The electron rich *β*-position of indole ring then attacks the electron deficient conjugated carbon-carbon double bond of **4** to afford **5**, followed by a hydrogen transfer to yield **6**. Finally **6** rearranged to give target compound **3a** and [Sbmim][HSO_4_] catalyzes the next cycle.

## Conclusions

In summary, we have reported an efficient and simple method for synthesis of a series of novel 1-aryl-3-(indol-3-yl)-3-(2-aryl-1,2,3-triazol-4-yl)propan-1-one using 1-aryl-3-(2-aryl-1,2,3-triazol-4-yl)propan-1-one instead of ordinary *α,β*-unsaturated ketones as substrate and employing Brønstedacidic ionic liquid [Sbmim][HSO_4_] as catalyst. The corresponding adducts were synthesized in short reaction times successfully and isolated in excellent yields. It is an important supplement to the existing methods for the synthesis of *β*-indolylketones. 

## Experimental

### General

All compounds were characterized by IR, ^1^H-NMR spectra and elemental analysis. The IR spectra were obtained as potassium bromide pellets with a FTS-40 spectrometer (BIO-RAD, U.S.A). The ^1^H-NMR spectra were obtained on a Varian Inova-400 spectrometer using CDCl_3_ or DMSO-d_6_ as solvent (as indicated under each entry below) and TMS as an internal standard, chemical shifts are given in ppm. Elemental analyses (C, H, N) were performed on a Perkin-Elmer Analyzer 2400. Melting points were determined using a Büchi B-540 instrument. All melting points are uncorrected. The Brønsted acid ionic liquid [Sbmim][HSO_4_] was synthesized according to a previous literature method [19]. 1-aryl-3-(2-aryl-1,2,3-triazol-4-yl)propan-1-one (**2**) was synthesized according to a previous literature report [21]. 

### General procedure for the synthesis of 1-aryl-3-(indol-3-yl)-3-(2-aryl-1,2,3-triazol-4-yl)propan-1-ones ***3a-3j***

A mixture of indole (2 mmol), 1-aryl-3-(2-aryl-1,2,3-triazol-4-yl)propan-1-one (2 mmol) and [sbmim][HSO_4_] (0.2 mmol) was heated at 80 °C in acetonitrile (10 mL) or 90 °C under solvent-free conditions for 1 h with stirring (Scheme 1). The completion of the reaction was monitored by TLC. After cooling, the reaction mixture was poured onto crushed ice (30 g). The resulting precipitate was ﬁltered under suction, and then recrystallized from ethanol to afford the pure product. The results are summarized in Table 2. Data of the compounds are shown below:

*1-Phenyl-3-(indol-3-yl)-3-(2-phenyl-1,2,3-triazol-4-yl)propan-1-one* (**3a**): Red powder. ^1^H-NMR (DMSO-*d_6_*) δ: 3.87 (dd, *J* = 6.0, 6.0 Hz, 1H, CH), 4.18 (dd, *J* = 8.4, 8.4 Hz, 1H, CH), 5.15 (t, 1H, *J* = 7.2 Hz, CH), 6.96–8.06 (m, 16H, ArH), 10.97 (s, 1H, NH); IR: ν 3342, 1680, 1594, 1457, 1357, 748 cm^–1^; Anal. Calcd. for C_25_H_20_N_4_O: C, 76.51; H, 5.14; N, 14.28. Found: C, 76.36; H, 5.18; N, 14.22.

*1-(4-Methylphenyl)-3-(indol-3-yl)-3-(2-phenyl-1,2,3-triazol-4-yl)propan-1-one* (**3b**): Light yellow powder. ^1^H-NMR (DMSO-*d_6_*) δ: 2.37 (s, 3H, CH_3_), 3.82 (dd, *J* = 6.0, 6.4 Hz, 1H, CH), 4.12 (dd, *J* = 8.4, 8.4 Hz, 1H, CH), 5.13 (t, 1H, *J* = 7.2 Hz, CH), 6.96–8.01 (m, 15H, ArH), 10.96 (s, 1H, NH); IR: ν 3359, 1674, 1595, 1456, 1337, 736 cm^–1^; Anal. Calcd. for C_26_H_22_N_4_O: C, 76.83; H, 5.46; N, 13.78. Found: C, 76.71; H, 5.51; N, 13.72.

*1-(4-Methoxylphenyl)-3-(indol-3-yl)-3-(2-phenyl-1,2,3-triazol-4-yl)propan-1-one* (**3c**): Light yellow powder. ^1^H-NMR (DMSO-*d_6_*) δ: 3.78 (dd, *J* = 6.4, 6.4 Hz, 1H, CH), 3.84 (s, 3H, OCH_3_) 4.08 (dd, *J* = 8.4, 8.8 Hz, 1H, CH), 5.12 (t, 1H, *J* = 7.2 Hz, CH), 6.95–8.04 (m, 15H, ArH), 10.95 (s, 1H, NH); IR: ν 3348, 2901, 1668, 1595, 1457, 1338, 735 cm^–1^; Anal. Calcd. for C_26_H_22_N_4_O_2_: C, 73.92; H, 5.25; N, 13.26. Found: C, 73.81 ; H, 5.32; N, 13.39.

*1-(4-Chlorophenyl)-3-(indol-3-yl)-3-(2-phenyl-1,2,3-triazol-4-yl)propan-1-one* (**3d**): Light yellow powder. ^1^H-NMR (DMSO-*d_6_*) δ: 3.85 (dd, *J* = 6.0, 6.0 Hz, 1H, CH), 4.17 (dd, *J* = 8.8, 8.8 Hz, 1H, CH), 5.12 (t, 1H, *J* = 7.2 Hz, CH), 6.96–8.17 (m, 15H, ArH), 10.97 (s, 1H, NH); IR: ν 3395, 1678, 1586, 1490, 1338, 737 cm^–1^; Anal. Calcd. for C_25_H_19_N_4_OCl: C, 70.34; H, 4.49; N, 13.12. Found: C, 70.48 ; H, 4.55; N, 13.01.

*1-(4-Bromophenyl)-3-(indol-3-yl)-3-(2-phenyl-1,2,3-triazol-4-yl)propan-1-one* (**3e**): Red powder. ^1^H-NMR (DMSO-*d_6_*) δ: 3.84 (dd, *J* = 6.0, 6.0 Hz, 1H, CH), 4.16 (dd, *J* = 8.4, 8.4 Hz, 1H, CH), 5.12 (t, 1H, *J* = 7.2 Hz, CH), 6.96–8.10 (m, 15H, ArH), 10.96 (s, 1H, NH); IR: ν 3389, 1677, 1583, 1491, 1338, 737 cm^–1^; Anal. Calcd. for C_25_H_19_N_4_OBr: C, 63.70; H, 4.06; N, 11.89. Found: C, 63.81; H, 4.11; N, 11.75.

*1-(2,4-Dichlorophenyl)-3-(5-bromoindol-3-yl)-3-(2-phenyl-1,2,3-triazol-4-yl)propan-1-one* (**3f**): Light yellow powder. ^1^H-NMR (DMSO-*d_6_*) δ: 3.77 (dd, *J* = 6.0, 6.4 Hz, 1H, CH), 4.04 (dd, *J* = 8.4, 8.8 Hz, 1H, CH), 5.03 (t, 1H, *J* = 7.6 Hz, CH), 7.16–8.06 ( m, 13H, ArH), 11.20 (s, 1H, NH); IR: ν 3323, 1689, 1581, 1461, 1340, 817, 753 cm^–1^; Anal. Calcd. for C_25_H_17_N_4_OCl_2_Br: C, 55.58; H, 3.17; N, 10.37. Found: C, 55.68; H, 3.10; N, 10.25.

*1-Phenyl-3-(2-methylindol-3-yl)-3-(2-phenyl-1,2,3-triazol-4-yl)propan-1-one* (**3g**): Red powder. ^1^H-NMR (400 MHz, DMSO-*d_6_*) δ: 2.44 (s, 3H, CH_3_), 3.81 (dd, *J* = 6.8, 6.8 Hz, 1H, CH), 4.25 (dd, *J* = 7.2, 7.6 Hz, 1H, CH), 5.09 (t, 1H, *J* = 7.6 Hz, CH), 6.85–8.01 (m, 15H, ArH), 10.84 (s, 1H, NH); IR (KBr): ν 3368, 2971, 1679, 1595, 1460, 1336, 752 cm^–1^; Anal. Calcd. for C_26_H_22_N_4_O: C, 76.83; H, 5.46; N, 13.78. Found: C, 76.91; H, 5.41; N, 13.89.

*1-Phenyl-3-(2-methylindol-3-yl)-3-(2-(4-bromophenyl)-1,2,3-triazol-4-yl)propan-1-one* (**3h**): Red powder. ^1^H-NMR (DMSO-*d_6_*) δ: 2.45 (s, 3H, CH_3_), 3.82 (dd, *J* = 6.8, 7.2 Hz, 1H, CH), 4.23 (dd, *J* = 7.2, 7.6 Hz, 1H, CH), 5.07 (t, 1H, *J* = 7.2 Hz, CH), 6.84–7.99 (m, 14H, ArH), 10.84 (s, 1H, NH); IR: ν 3378, 2898, 1675, 1593, 1489, 739 cm^–1^; Anal. Calcd. for C_26_H_21_N_4_OBr: C, 64.34; H, 4.36; N, 11.54. Found: C, 64.45; H, 4.30; N, 11.72.

*1-(2-Chlorophenyl)-3-(2-methylindol-3-yl)-3-(2-phenyl-1,2,3-triazol-4-yl)propan-1-one* (**3i**): Red powder. ^1^H-NMR (DMSO-*d_6_*) δ: 2.39 (s, 3H, CH_3_), 3.70 (dd, *J* = 6.8, 6.8 Hz, 1H, CH), 4.18 (dd, *J* = 8.0, 8.4 Hz, 1H, CH), 5.02 (t, 1H, *J* = 7.6 Hz, CH), 6.83–7.94 (m, 14H, ArH), 10.88 (s, 1H, NH); IR: ν 3380, 1692, 1588, 1460, 1354, 746 cm^–1^; Anal. Calcd. for C_26_H_21_N_4_OCl: C, 70.82; H, 4.80; N, 12.71. Found: C, 70.65; H, 4.83; N, 12.87.

*1-(4-Methoxylphenyl)-3-(1-methylindol-3-yl)-3-(2-phenyl-1,2,3-triazol-4-yl)propan-1-one* (**3j**): Light yellow powder. ^1^H NMR (DMSO-*d_6_*) δ: 3.72 (s, 3H, CH_3_) 3.76 (dd, *J* = 6.0, 6.0 Hz, 1H, CH), 3.84 (s, 3H, OCH_3_), 4.09 (dd, *J* = 8.4, 8.8 Hz, 1H, CH), 5.11 (t, 1H, *J* = 7.2 Hz, CH), 6.99–8.03 (m, 15H, ArH); IR: ν 3348, 2909, 1682, 1596, 1464, 731 cm^–1^; Anal. Calcd. for C_27_H_24_N_4_O_2_: C, 74.19; H, 5.54; N, 12.84. Found: C, 74.35; H, 5.59; N, 12.99.
